# Secondary Immune Deficiency and Primary Immune Deficiency Crossovers: Hematological Malignancies and Autoimmune Diseases

**DOI:** 10.3389/fimmu.2022.928062

**Published:** 2022-07-18

**Authors:** Mark Ballow, Silvia Sánchez-Ramón, Jolan E. Walter

**Affiliations:** ^1^ Division of Pediatric Allergy and Immunology, Department of Pediatrics, Morsani College of Medicine, University of South Florida at Johns Hopkins All Children’s Hospital, St Petersburg, FL, United States; ^2^ Department of Immunology, Hospital Clínico San Carlos, Instituto de Medicina del Laboratorio (IML), Complutense University of Madrid, Madrid, Spain; ^3^ Division of Pediatric Allergy and Immunology, Massachusetts General Hospital, Harvard Medical School, Boston, MA, United States

**Keywords:** autoimmune disease, hematological malignancy, immune deficiency, primary immunodeficiency, secondary immunodeficiency

## Abstract

Primary immunodeficiencies (PIDs), a heterogenous group of inborn errors of immunity, are predetermined at birth but may evolve with age, leading to a variable clinical and laboratory presentation. In contrast, secondary immunodeficiencies (SIDs) are acquired declines of immune cell counts and or/function. The most common type of SID is a decreased antibody level occurring as a consequence of extrinsic influences, such as an underlying condition or a side effect of some medications used to treat hematological malignancies and autoimmune disorders. Paradoxically, immune deficiencies initially attributed to secondary causes may partly be due to an underlying PID. Therefore, in the era of immune-modulating biologicals, distinguishing between primary and secondary antibody deficiencies is of great importance. It can be difficult to unravel the relationship between PID, SID and hematological malignancy or autoimmunity in the clinical setting. This review explores SID and PID crossovers and discusses challenges to diagnosis and treatment strategies. The case of an immunodeficient patient with follicular lymphoma treated with rituximab illustrates how SID in the setting of hematological cancer can mask an underlying PID, and highlights the importance of screening such patients. The risk of hematological cancer is increased in PID: for example, lymphomas in PID may be driven by infections such as Epstein-Barr virus, and germline mutations associated with PID are enriched among patients with diffuse large B-cell lymphoma. Clues suggesting an increased risk of hematological malignancy in patients with common variable immune deficiency (CVID) are provided, as well as pointers for distinguishing PID versus SID in lymphoma patients. Two cases of patients with autoimmune disorders illustrate how an apparent rituximab-induced antibody deficiency can be connected to an underlying PID. We highlight that PID is increasingly recognized among patients with autoimmune cytopenias, and provide guidance on how to identify PID and distinguish it from SID in such patients. Overall, healthcare professionals encountering patients with malignancy and/or autoimmunity who have post-treatment complications of antibody deficiencies or other immune abnormalities need to be aware of the possibility of PID or SID and how to differentiate them.

## Introduction

Primary immunodeficiencies (PIDs), a heterogenous group of inborn errors of immunity, are predetermined at birth but may evolve with age, leading to a variable clinical and laboratory presentation. They are classified by the International Union of Immunological Societies according to the main immune compartments and function affected into 10 different classes, that include combined immunodeficiencies (T- and B-cell defects) with or without syndromic features, versus antibody deficiencies (B-cell defects), or immune dysregulation (impaired regulation and/or abnormal activation of immune subsets) (1).

In contrast, secondary immunodeficiencies (SIDs) are acquired declines of immune cell counts and or/function. The most common type of SID is a decreased antibody level occurring due to an underlying condition or as a side effect of medications used to treat hematological malignancies and autoimmune disorders (2, 3). Paradoxically, immune deficiencies initially attributed to secondary causes may partly be due to an underlying PID. In the era of immune-modulating biologicals, distinguishing between primary and secondary antibody deficiencies is of great importance.

Both PIDs and SIDs can be associated with infections, immune dysregulation, autoimmune disorders, lymphoproliferation and malignancy, and it can be difficult to unravel the relationship between these disorders in the clinical setting (4). A study looking at the initial clinical manifestations of confirmed PID found that most patients (68%) had a history of infection, but an exclusive focus on infection-centered manifestations would have missed a substantial number of patients who initially presented with other findings such as immune dysregulation (5). It is known that patients with PID are at increased risk of hematological malignancy and autoimmune disorders (6, 7). On the other hand, SID can develop as a consequence of B-cell lymphoproliferative diseases such as multiple myeloma and chronic lymphocytic leukemia (CLL) (3, 8). Furthermore, immune modulators such as B-cell depleting therapies and checkpoint inhibitors used to treat hematological and autoimmune disorders can cause transient or prolonged SID (9, 10).

This paper uses case histories to explore clinical crossovers between PID, SID and hematological malignancies or autoimmune disorders, and the challenges that can arise in differentiating PID from SID and in providing suitable treatment strategies.

## Crossovers Between SID, PID and B-Cell Lymphoproliferative Disease

### Case History: SID Masking PID in a Patient With Hematological Cancer

The case of a patient with follicular lymphoma and immunodeficiency shows how challenging the diagnosis of PID versus SID can be for physicians.

A 67-year-old female patient presented to the Emergency department in 2003 with severe abdominal pain. She had a history of recurrent respiratory infections from childhood, one episode of pneumonia, and hypertension. Physical examination and imaging revealed multiple lymphadenopathies in several regions, including the lung and bone marrow, and an inguinal node biopsy found stage IV-B follicular lymphoma. She underwent five cycles of chemotherapy with fludarabine, cyclophosphamide and rituximab (FCR) and achieved complete remission in 2004. At her initial presentation she also had an osteolytic cavity in the L4 vertebral cavity, with a soft tissue mass that was diagnosed as a peripheral nerve sheath tumor at L4–L5, for which she underwent radiation therapy.

She experienced a recurrence of lymphoma in 2006 (i.e., within 2 years of diagnosis, which is an unfavorable prognostic sign) and received rituximab maintenance therapy for 2 years, again leading to complete remission. She was referred in 2016 to the Immunology department due to severe panhypogammaglobulinemia (low IgG, IgA, IgM) with full absence of B cells in the setting of lymphoma and severe infections. She was already receiving intermittent immunoglobulin replacement therapy (IgRT). An immunological work-up showed that her immunological profile was marked by a predominantly humoral defect (panhypogammaglobulinemia), with normal T cell and neutrophil counts. Regarding functional (specific) antibody responses, after relevant immunizations the patient had detectable anti-Pneumococcus (Pneumo-23) and anti-tetanus toxoid antibodies while on IgRT. However, these levels can be normal in patients who are receiving intravenous immunoglobulin therapy because the relevant antibodies are present in the infused product, therefore it is difficult to distinguish the patient’s own response to the vaccine. In contrast, the response to anti-*Salmonella typhi* was low. This was a useful finding, because antibodies to *S. typhi* are undetectable in intravenous immunoglobulin products and therefore do not influence plasma concentrations. Thus, exposure to *S. typhi* antigen can help to evaluate functional antibody responses in patients on IgRT (11). Overall, this suggested the presence of both quantitative (panhypogammaglobulinemia) and qualitative (low vaccine titer) antibody deficiency.

The patient’s overall clinical course has varied over time, with episodes of recurrent pneumonia, herpes zoster, bacteremia, two second primary neoplasia, and severe malabsorption. She has had a lack of humoral reconstitution despite being in remission since 2008. Due to chronic diarrhea, severe malnutrition and low bodyweight, she underwent a duodenal endoscopy, which revealed marked villous atrophy (Marsh grade 3b). A biopsy showed intraepithelial lymphocytes and no plasma cells. On examining the combined proportion of total intraepithelial lymphocytes, there were no TCRγ/δ cells (i.e., a celiac-like phenotype), normal expression of CD3 (T cells) and CD103 (tissue-resident memory T cells), and low iNKT (which reflects mucosal damage). The overall picture was of a severe celiac-like enteropathy with a distinctive immunophenotype. The presence of herpes zoster infections and enteropathy points toward immune dysfunction and dysregulation of T cells. In combination with the humoral immunodeficiency, this patient now displayed features of combined immunodeficiency with immune dysregulation. Follow-up of the patient’s lymphoma with assessment of serum free light chains found that kappa and lambda were almost undetectable until June 2021 when there was an abrupt increase in kappa chains. She is now being evaluated for recurrence again, 18 years after the onset of initial disease.

In summary, a 67-year-old woman presented with follicular lymphoma, recurrent respiratory infections since childhood, malabsorption, recurrence of lymphoma within 2 years of onset, very low immunoglobulin levels, a lack of B-cell reconstitution after chemotherapy, non-infectious complication (enteropathy), two primary second neoplasia, and severe recurrent infections despite immunoglobulin therapy suggestive of additional T-cell dysfunction.

Exome analysis revealed a homozygous LRBA variant, (LRBA):c.3076C>T (p.Gln1026Ter). There was functional evidence that CTLA4 expression was absent on the surface of activated T cells. LRBA deficiency is autosomal recessive associated with a specific PID, common variable immune deficiency (CVID), with a spectrum of mutations and highly variable clinical and immunologic characteristics (12). Overall, the combined immunodeficiency phenotype with enteropathy and malignancy might suggest a defect within the spectrum of regulatory T-cell disorders (Tregopathy), such as IPEX-like, CTLA4, and LRBA deficiencies (13–15). Thus, we cannot rule out that this follicular lymphoma patient might actually have an underlying PID rather than a malignancy and/or drug (anti-CD20) induced SID.

The case illustrates how SID in the setting of hematological cancer can mask an underlying PID. It is therefore important to perform immunodeficiency screening at cancer onset. Further discussion of this aspect in the following sections will focus primarily on CVID, one of the more common PIDs.

### Common Variable Immune Deficiency and Hematological Cancer: Screening

The risk of cancer is increased in patients with CVID (16–18) and a delay in diagnosing CVID is unfavorable for cancer prognosis with respect to infection risk and suboptimal response to therapy secondary to increased toxicity and recurrence (17). Patients with cancer and laboratory evidence of immune abnormalities, with or without infections, should be screened for CVID and other immune deficiencies before therapy is initiated. This will allow treating physicians to modify management based on the underlying PID.

Screening for cancer in patients with known CVID would appear sensible; however, it can be challenging to distinguish cancer from lymphoproliferative processes. Other comorbidities such as inflammation can also confound the picture. There are no consensus guidelines for cancer screening and follow-up in these patients. In addition, some authors have proposed a specific histologic subclassification of PID-associated lymphoma (19). The approach used should be mindful about whether or not biopsies and scans are needed to establish the diagnosis. A basic immunological work-up is important before starting cancer therapy.

Given that either face of the problem, immunodeficiency or hematological malignancy, may be seen first, it is important that an interdisciplinary approach is used for the diagnosis and management of these patients (3, 20–22).

### PID Carries an Increased Risk of Hematological Cancer

Immunodeficiency is associated with a high risk of cancer. PID carries a 1.4–5 fold increased risk of cancer compared with the general population based on registry studies, although the absolute rate is still low (6, 23). The rate of malignancy in the subset of CVID patients is approximately 10% (6, 23, 24), and lymphoproliferative syndromes are 10-fold more common in CVID compared with the general population (25). This is relevant because cancer is the second most common cause of mortality in PID patients (after infection) (26). The pooled prevalence of lymphoma is low at 4.1%, based on meta-analyses and registry-based studies, and that of gastric adenocarcinoma is 1.5% (16, 25).

Looking specifically at patients with hematological cancer (one of the ten most common forms of cancer) and lymphoproliferative syndromes, it is enriched in immunocompromised individuals, with germline mutations described in 1–18% of cases in preliminary studies (27). It is estimated that 20% of human cancers are associated with chronic or latent infections (28, 29), which are also more frequent in immunodeficient patients. Classically, 10% of hematological cancers in children are associated with congenital syndromes; however, in a recent study, whole-exome sequencing revealed genetically confirmed PID in up to 62% of children with lymphoproliferative disorders (30). This has clinical and prognostic implications, including for treatment decisions. If lymphoproliferative disorders are similarly enriched in PID patients, this raises the question of the real magnitude of PID in global cancer and non-malignant hematological lymphoproliferative disorders.

### CVID Within the Cancer Pool

Over the last 20 years we have gained insight into the burden of infection-driven cancers, and in particular the importance of chronic infections, with *Helicobacter pylori*, human papilloma virus, and hepatitis B and C estimated to cause 16% of all cancers worldwide (31). Several studies have highlighted that lymphoma in PID is commonly caused by Epstein-Barr virus infections (19, 32, 33).

**Figure 1:**
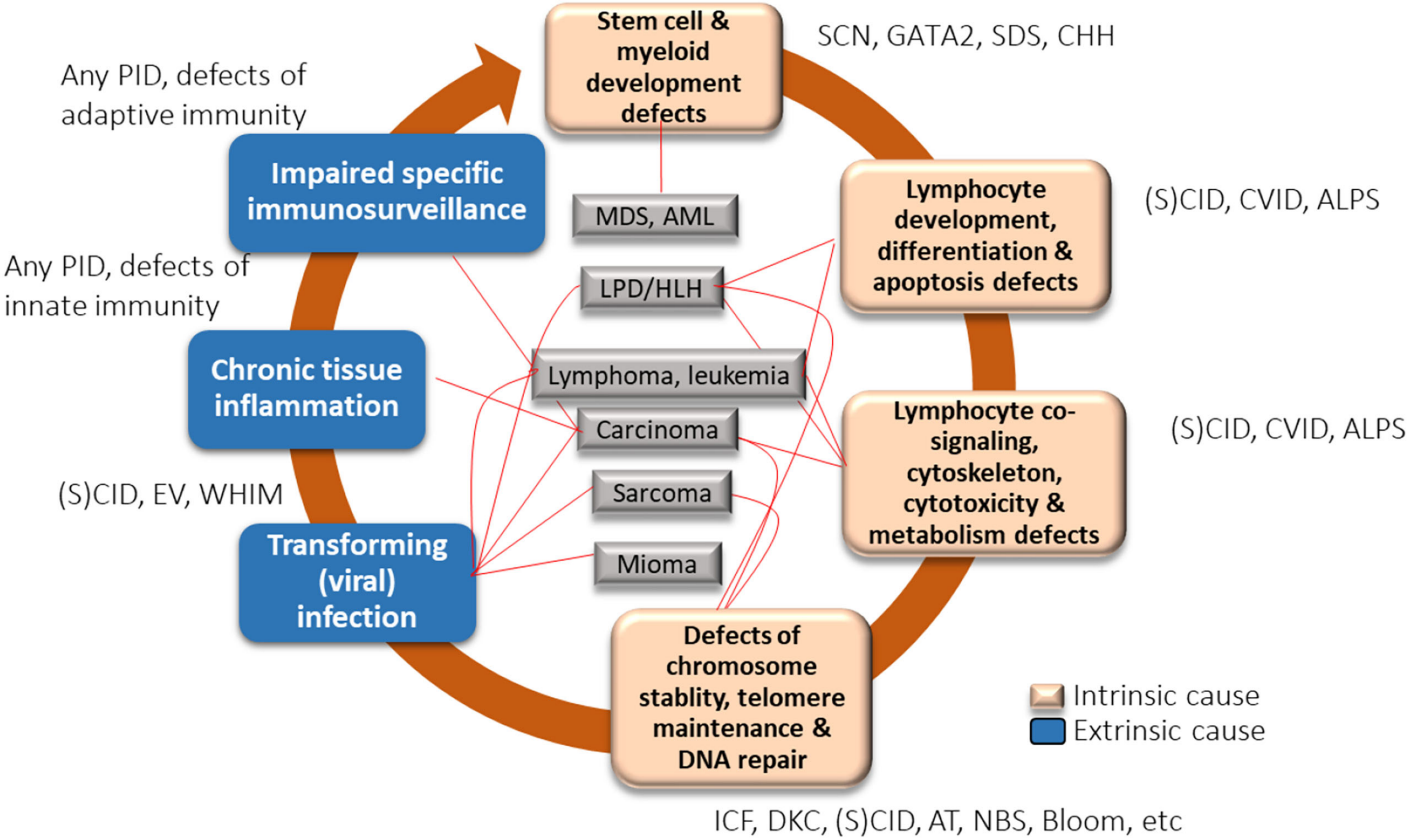
Factors implicated in carcinogenesis in common variable immune deficiency (CVID) [created with data from Hauck et al., 2018 (34)]. ALPS, autoimmune lymphoproliferative syndrome; AML, acute myelogenous leukemia; CVID, common variable immune deficiency; DKC, dyskeratosis congenita; EV, epidermodysplasia verruciformis; IBD, inflammatory bowel disease; ICF, immunodeficiency with centromeric instability and facial anomalies; LPD, lymphoproliferative disorder; MDS, myelodysplastic syndrome; PID, primary immunodeficiency; (S)CID, (severe) combined immunodeficiency; SDS, Shwachman-Diamond syndrome; SM, smooth muscle tumor; WHIM, warts, hypogammaglobulinemia, immunodeficiency, and myelokathexis.

Cancer is a complex multifactorial process, with both intrinsic and extrinsic factors implicated in its pathogenesis. Various factors implicated in carcinogenesis in CVID are summarized in **Figure 1** (34). Intrinsic factors include a genetically coded defect in the machinery of DNA repair, defects in T- and B-cell receptor (VDJ) or class switch recombination (Ig isotypes) and somatic hypermutation, and defects in processes involved in B-cell maturation and costimulatory signaling. Extrinsic factors include impaired immunosurveillance, dysbiosis, persistent mucosal inflammation, and decreased clearance of oncogenic viral and bacterial infections, including *H. pylori* infection and transforming viral infections such as Epstein-Barr infection.

A systematic review and meta-analysis of the prevalence of malignancy in CVID patients, based on 48 studies, found a pooled prevalence of malignancy of 8.6% (790 cases among 8123 CVID patients) (16). Lymphoma was the most frequent malignancy (4% of patients; 40% of cancers).

#### Non-Hodgkin’s Lymphoma and Germline Mutations: PID?

Diffuse large B-cell lymphoma (DLBCL) is the most common hematological malignancy. The search for germline mutations has added a layer of complexity. Based on a comprehensive review of this topic, among 626 genes in which somatic mutations have been found in DLBCL, 24 have also been observed to carry pathogenic mutations in germline form in DLBCL patients (27). One-third of the somatically mutated genes found in DLBCL were already linked to primary immunodeficiencies (e.g. *ATM, DCLRE1C, PRKDC, IFNGR1, PIK3CD, PIK3R1, STAT3, FAS*). A phenotypic characterization of 193 germline mutations among 211 somatically mutated genes in DLBCL found that immunodeficiency was present in 17% of cases (27). These preliminary data suggest that inherited mutations in DLBCL can have other specific phenotypes such as PID, which together with environmental factors can lead to lymphoma.

Thus, it is possible that genetic tools may be able to identify PID in the presence of lymphoma. As has been observed in other malignancies, an increase in genetic screening will reveal mutations in germlines, which were originally found in somatically mutated lymphomas.

### Facing a CVID Patient: Clues for Hematological Malignancy

When managing a patient with CVID, there are potential clues that can point towards a risk of lymphoma. Several studies have compared CVID patients with and without malignancy to search for predictive markers or clinical profiles (**Table 1**) (16, 18, 19, 25, 26, 35–39). Potential risk factors that were identified included non-malignant lymphoproliferative disorders, lymphadenopathy, autoimmune cytopenia, enteropathy, altered IgM levels, older age at CVID diagnosis, sex, late-onset combined immunodeficiency, germline genetic mutations (i.e. *CTLA4, PI3KR1/PI3KCD, LRBA*), and Epstein-Barr virus susceptibility.

**Table 1:** Potential predictors of hematological malignancy risk in patients with common variable immune deficiency (CVID).

Pre-existing or concomitant lymphoproliferative disease (2.5-fold increased risk of lymphoma; p=0.005) (25)
Longstanding lymphadenopathy or splenomegaly (16, 19, 26)
Autoimmune manifestations, including cytopenia, in some (16, 18, 19, 25) but not all (25) studies
Non-infectious gastrointestinal involvement/enteropathy in some (16, 19) but not all (25) studies
High IgM levels at diagnosis of CVID in some studies (16, 26, 35); low IgM in other studies (19)
Greater age at CVID diagnosis (25)
Female sex (35); Male sex (19)
Late-onset combined immunodeficiency (loCID) phenotype (36)
Mutations in *CTLA4*, *BACH2*, *TNFSR13B*, PIK3CD, *PIK3R1, CD27, CD70, NFkB, CD19, TWEAK, CD21, ICOS, IRF2BP2* (37, 38)
EBV susceptibility (39)

A meta-analysis of nine studies that compared demographic, clinical and immunologic characteristics between CVID patients with (n=26) or without (n=161) malignancy, found that patients with malignancy were significantly older, had higher levels of IgG, IgA and IgM (although actual levels were lower than normal), and higher frequencies of autoimmunity and malabsorption (16). Higher IgM was associated with lymphoid hyperplasia and lymphoid malignancy. Polyautoimmunity was present in half the patients. Overall, there are several clinical and laboratory clues that should alert the clinician to hematological malignancy in a patient with CVID, and especially PIRD and late-onset CID groups, which it is important to distinguish from CVID.

### Pointers Towards PID Versus SID in Lymphoma Patients

Potential markers that point towards PID rather than SID in patients with hematological malignancy are summarized in **Table 2**. For example, a past medical history of recurrent/severe infections or autoimmune disease and lack of immune reconstitution after therapy suggests PID. In-depth immune phenotyping and antibody testing before initiation of treatment for lymphoma could distinguish PID from SID. Genetic studies investigating not only somatic but also germline mutations associated with lymphoma can be of help in patients for whom there is a concern about underlying PID.

**Table 2:** Variables that can help differentiate between PID and SID in patients with hematological malignancy.

Item	PID	SID
**Recurrent/severe infections, autoimmune diseases, enteropathy**	**Past** medical history Family history	**After** cancer and/or cancer therapy, other causes
**Immunological variables at lymphoma diagnosis**	Low IgG/IgA ( <2SD) Low levels of natural antibodies Low antibody responses at cancer diagnosis (primary and secondary responses) Very low serum free kappa/lambda (specific for CVID) Defect in memory B-cell phenotype	Normal/low secondary responses
**B-cell reconstitution after therapy**	No	Rare but **possible**
**Response to cancer therapy**	Toxicity, infections, second primary cancer, cancer recurrence	
**Genetic studies**	Germline mutations associated with lymphoma/PID	
**Preventive strategies**	Active surveillance of other complications (clinical, imaging, endoscopy), choice of cancer immunotherapy, PCR/IH search for oncogenic viruses in blood or tissues	

CVID, common variable immune deficiency; IH, immunohistochemistry; PCR, polymerase chain reaction; PID, primary immunodeficiency; SID, secondary immunodeficiency.

## Crossovers Between SID, PID and Autoimmunity

### Case History 1: Rituximab-Associated SID Versus PID in an Adult With cITP

The case of a 52-year-old businessman who had been treated within various specialties over a period of many years illustrates the difficulties in differentiating PID from SID in patients with autoimmune disorders.

At age 11 years, he was found to have a few enlarged lymph nodes. By age 27 years, he was diagnosed with chronic immune thrombocytopenia (cITP) after his platelet count dropped to critically low level (<5,000 count/μL), for which he was given high-dose steroid treatment. He did well during follow-up by the hematologist. When aged 30 years, he developed a chronic cough; imaging revealed mild bronchiectasis and a few small pulmonary nodules, and a pulmonologist was included into his clinical management team. At age 33 years, he was referred for submandibular and transbronchial lymph node biopsy, which found non-malignant lymphoid hyperplasia. At age 46 years he was admitted to hospital with a platelet count of 0; he was found to have splenomegaly (18 cm), but a bone marrow aspiration did not show abnormalities. Jointly, the hematologist and pulmonologist decided to treat him acutely with steroids and high dose intravenous immunoglobulin (IVIg) because of the low platelet count, followed by B-cell depleting rituximab (anti-CD20 therapy) for the history of cITP. He had an excellent response and his platelet count normalized. In addition, there was “bystander improvement” in his lung nodules, which surprised the pulmonologist. It was agreed that rituximab was a useful medication for immune dysregulation in this patient.

A second team of specialists became involved in the period after he had received rituximab therapy. He developed “new onset” hypogammaglobulinemia, leading to the involvement of an immunologist; he had an excellent response to IgRT. His cough improved and his energy levels increased. However, he experienced intermittent upper respiratory tract infections and sinus disease, for which he was treated with antibiotics and monitored by ear, nose and throat (ENT) and infectious disease specialists.

At age 48 years, the pulmonary nodules enlarged again, his platelet count dropped, and his spleen increased in size, and therefore he was given a second dose of rituximab. This was followed by a third dose at age 51 years because of relapsing symptoms. During this period not all aspects of his health improved. He needed repeated antibiotic treatment, even though his IgRT dose was increased to almost 1g/kg/month. He experienced *Salmonella*, *Clostridium difficile* and chronic *Hemophilus influenzae* type B infections. Since the initial IgRT replacement the Immunology team has been monitoring his immune function. He was found to have no detectable IgA and IgM (even at 1 year after receiving his first rituximab treatment), and he developed progressive B and T cell lymphopenia (including low naïve CD4 T-cell count and ratio of naïve to memory CD4 T cells). Genetic testing revealed a heterozygous pathogenic mutation (c.967_979del, p.Leu323Serfs*51) in the PHD1 domain of *AIRE*. Based on the information provided with the Invitae Genetic Test Kit “this variant is also known as c.964del13, c.967_979del13, p.C322del13, or c.1094_1106del” in the literature (Invitae Gene Testing). A paper by Oftedal et al. suggests that this mutation results in a partial dominant effect (40). The reported patient in the published cohort had pernicious anemia and no anti-cytokine antibodies. Genetic testing for a PID panel also showed *ORAI1* heterozygous mutation, as well as *NFAT5* and *SMARCAL1* VUS of unknown significance. The finding of a pathogenic AIRE variant is curious, as in addition to repeated infections, the patient has a history of non-infectious complications, including alopecia, fatty liver, and hypogonadism. Overall, it had initially been thought that he had antibody deficiency induced by rituximab, i.e., SID. However, over the course of his repeated treatments it became apparent that there is likely a PID component to his disease.

This case raises several questions. Firstly, can we say for certain what is wrong with the patient - PID or SID? He could have SID as the result of medical therapy (rituximab) or chronic disease. However, the predisposition to infections and evidence of prolonged autoimmune disease and immune dysregulation pointed towards PID. Secondly, how long should he receive replacement immunoglobulins and at what dosing schedule? Long-term IgRT may not be needed in patients with SID in whom treatment leads to B-cell immune reconstitution, whereas patients with PID generally need lifelong replacement therapy. In this patient, a decision had to be made about whether to give low-dose (0.5 g/kg) IgRT, or to add anti-inflammatory therapy to a high dose (1.0 g/kg). Thirdly, when and how should immunomodulation be performed? The patient is already receiving high-dose IgRT. Although rituximab has shown some efficacy he has relapses, therefore the quest for targeted biologicals should be continued to optimize his treatment course.

### Case History 2: Rituximab-Associated SID Versus PID in an Adolescent With Hemolytic Anemia

The second educational case depicts an adolescent with cytopenia, that combines the history of several patients (10). The patient presented at age 16 years with a history of Coombs-positive hemolytic anemia and lymphopenia. He was started on high-dose Ig and steroids but did not have an adequate response. He was then given rituximab as second-line therapy, which resulted in a partial response. Around the age of 17 years, he started developing infections, and 6 months after receiving the rituximab he was found to have low IgG, assumed to be SID, and was given IgRT. His platelet count had also decreased, with an autoimmune origin, and therefore he was diagnosed with ITP; since it was in combination with prior autoimmune hemolytic anemia (AIHA), he was categorized as one with Evans syndrome (multilineal cytopenia). With the increased awareness of PIDs among patients with Evans Syndrome, the concern was raised that the patient may have an underlying PID and not just a rituximab-induced hypogammaglobulinemia (SID). Tests showed increased “double negative” TCRαβ+CD4-CD8- T (DNT) cells and a pathogenic *FAS* variant, which confirmed a diagnosis of autoimmune lymphoproliferative syndrome (ALPS). Treatment with an mTOR inhibitor led to a successful response and remission.

This case illustrates that the initial assumption of a secondary antibody deficiency due to rituximab later evolved into a PID diagnosis of a ALPS. Understanding the underlying genetic defect and mechanism helped to differentiate SID and PID (10).

### Autoimmune Complications in Patients With PID

As illustrated by Case 1 above, many patients with PIDs may be managed by hematology, pulmonary, or gastrointestinal specialists as well as immunology departments. It is important to engage multidisciplinary teams in the evaluation and care of these patients.

In patients with PID, the treatment of infection is relatively straightforward and can be managed with antibiotics and IgRT. However, non-infectious complications, such as autoimmunity, inflammation, lymphoproliferation and malignancy, and severe allergies, can be difficult to treat with conventional therapies. Autoimmune complications may precede infection and can form a cluster of different diseases (e.g., ITP, hemolytic anemia, pulmonary nodules, enteropathy).

It is important to consider autoimmune complications in patients with PID. A large study showed that survival of PID patients with autoimmune complications (n=572) was reduced by 15% by age 80 years, compared to PID patients without such complications (n=1611) (41). We also know that many PID patients have autoimmune disorders as an initial presenting manifestation. An ESID Registry study involving more than 16,000 patients with PID found that almost 1 in 5 patients (18%) had evidence of immune dysregulation such as cytopenia or enteropathy at their initial presentation (5). Furthermore, these patients may have received biological therapies before full immune and genetic evaluation has been completed.

Autoimmune cytopenia is common across all PIDs. Notably, the primary immune dysregulation disorder (PIRD) group is highly enriched in autoimmunity overlapping with PID. Many PIRD patients have multiple autoimmune disorders; for example, 79% of those with LRBA deficiency have multi-autoimmune disorders (42). The first clinical symptom in PIRD is often autoimmune cytopenia, as seen in 33% of those with CTLA4 and 42% of those with LRBA deficiencies, which both present clinically as CVID variants with non-infectious complications (42, 43). The recently introduced “immune deficiency and dysregulation activity” (IDDA) scoring system may help to identify severe and/or treatment resistant cases and point to an underlying PIRD (44). Importantly, large cohort studies of specific genetic defects are now available; therefore, if a physician encounters a patient with a unique genetic entity plus autoimmune complications, information on these cohorts can be accessed to look for treatment outcomes which could guide the management of the patient (45).

Beyond PIRDs, many other immune gene defects have been linked to immune dysregulation, and this group of patients also often have a clinical phenotype of variable autoimmune disorders, with cytopenia being the most common (45).

#### Primary Immune Dysregulation Disorder (PIRD): Phenotypes and Diagnosis

The PIRD group of disorders includes combined clinical features of CVID, ALPS, profound or late onset combined immunodeficiency disorder (p-CID/lo-CID), and IPEX-like disease (immune dysregulation, polyendocrinopathy, enteropathy, X-linked syndrome) (45). Chandrakasan et al. reviewed PIRD clinical phenotypes and categorized them based on the dominant features of clinical presentation as ALPS-like, IPEX-like and CVID-like phenotypes (46). Immunoglobulin levels vary between the categories. A patient with an ALPS-like phenotype may have normal IgG levels and present only with immune cytopenia or lymphoproliferation, and therefore could mistakenly be assumed not to have PID. However, once they are treated with rituximab, the underlying immunodeficiency may be unmasked (as presented in Case 2). In contrast, patients with more of a CVID-like phenotype will have a low IgG level, which would immediately suggest a PID, such as antibody deficiency syndrome. However, if they initially present with cytopenia and IgG levels are not tested, the CVID-like phenotype could be missed and may only become evident after they have received biological therapies. For clinicians managing hematological malignancy in a patient with CVID features, it is important to distinguish PIRD and late-onset CID from CVID.

Given the variable etiologies for autoimmune cytopenia, how could we expedite the early diagnosis of PIRD patients? Extensive immune phenotyping should be undertaken (**Table 3**). Immunoglobulin levels are important; however, IgG levels are not always low in the initial stages. In addition to quantitative IgG, IgA, and IgM antibody levels, qualitative/functional antibody responses such as vaccine titers (e.g., tetanus, 23 serotypes of pneumococcus) and lymphocyte subsets should be evaluated. Low-switched memory B cells may be seen in CID and CVID-like presentations, while increased DNT cells point towards the ALPS-like phenotype, and regulatory T-cell abnormalities suggest the IPEX-like group. In addition, unique development abnormalities of immune cells can be particularly helpful in PIRD patients. For example, transitional B cells are increased markedly in in symptomatic patients with activated PI3Kδ syndrome (APDS) before optimal therapy. Many CVID patients have expansion of CD19^hi^CD21^lo^ B cells (also coined as double negative or age-associated B cells) and follicular helper T cells. These unique compartments may change with time and could be useful for both diagnosis and monitoring of response to therapy in PIRD.

**Table 3:** Diagnosing primary immune dysregulation disorders (PIRDs).

Immune phenotyping
Immunoglobulin levels (can be normal)*
Vaccine titers*
Lymphocyte subsets (T, B, NK)*
Unique developmental stages of immune cells: -Transitional B cells increased (APDS) -Low switched memory B cells (CID, CVID-like group)* -Increased double negative (TCR αβ+ CD4- CD8- (DN)) (ALPS-like group) -Regulatory T cell abnormalities (IPEX-like group) -CD19^hi^21^low^ B cells (age-associated B cells) and Tfh cells (monitoring)
**Genetic evaluation**
Genes associated with PID
**Functional assays**
May be needed for confirmation for VUS

ALPS, autoimmune lymphoproliferative syndrome; APDS, activated PI3Kδ syndrome; CID, combined immunodeficiency; CVID, common variable immune deficiency; IEI, inborn error of metabolism; IPEX, immune dysregulation, polyendocrinopathy, enteropathy, X-linked (syndrome); PID, primary immunodeficiency.

*Recommended evaluation before and after cancer treatment with biological agents.

Some healthcare providers, particularly those based in community care, may find it difficult to access laboratory platforms for extensive immune phenotyping. In such cases, genetic evaluations could be performed to search for genes associated with PID to identify those patients who may require further evaluation. Finally, confirmatory functional assays may be necessary in case of variants of uncertain significance.

**Figure 2:**
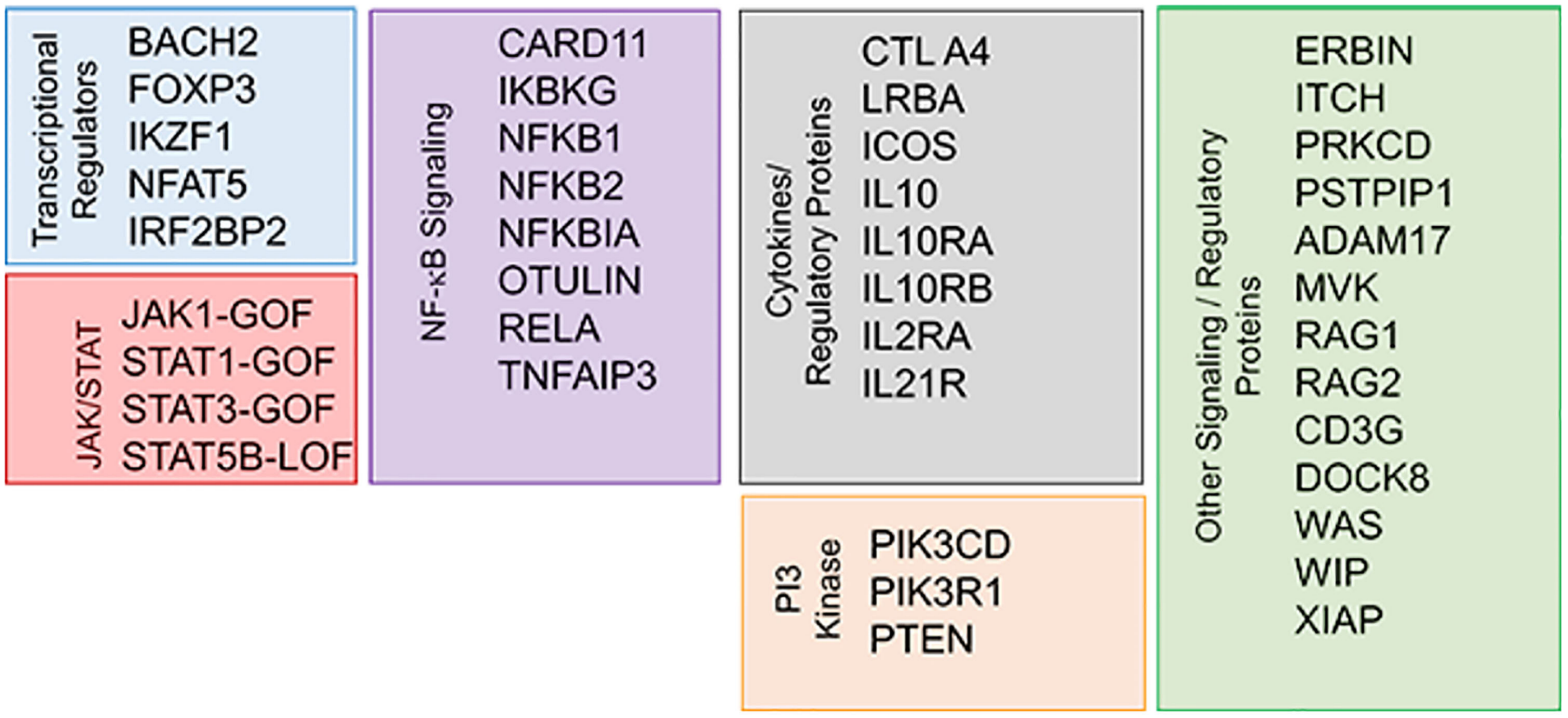
Genetic defects associated with primary immune dysregulation disorder (PIRD) (46, 47). Reproduced with permission from (47).

Genetic defects associated with PIRD are summarized in **Figure 2** (46, 47). Many of these defects are autosomal dominant (AD) by inheritance, meaning that even one abnormal allele can cause disease. Examples of AD genes in CVID and/or PIRDs include *NFKB1* (the most common cause of the CVID phenotype (48), *NFKB2, CTLA4*, *PIK3CD*, *PIK3R1*, *PCLG2*, *RAC2* (49–51). Genes commonly known to be associated with severe combined immunodeficiency (SCID), such as *RAG1* and *RAG2*, are increasingly recognized among older adults with CID and autoimmunity (52).

### Underlying PID in Patients With Autoimmune Cytopenia

We have discussed evidence that autoimmune disease, especially cytopenias, are common features of PID. On the other side of the coin, there is emerging understanding that PID is common in autoimmune cytopenia cohorts, especially among those with Evans syndrome where 40% of cases are PID (53). In a retrospective study of 154 children, 11% of patients with autoimmune cytopenia had PID, and 58% of PID cases had a confirmed monogenic cause (54). Based on the retrospective nature of this study, this is likely an underestimation. PID was diagnosed 3 years later than autoimmune cytopenia, except in patients with partial DiGeorge syndrome (pDGS). The study also looked for risk factors for PID in autoimmune cytopenia and found a greater frequency of splenomegaly and short stature, recurrent/chronic infections, low T cells (CD3, CD8) and immunoglobulins (IgG, IgA), and a higher prevalence of autoantibodies to erythrocytes, platelets, and neutrophils. Finally, the PID group with autoimmune cytopenia were more likely to fail first-line treatment (54).

**Figure 3:**
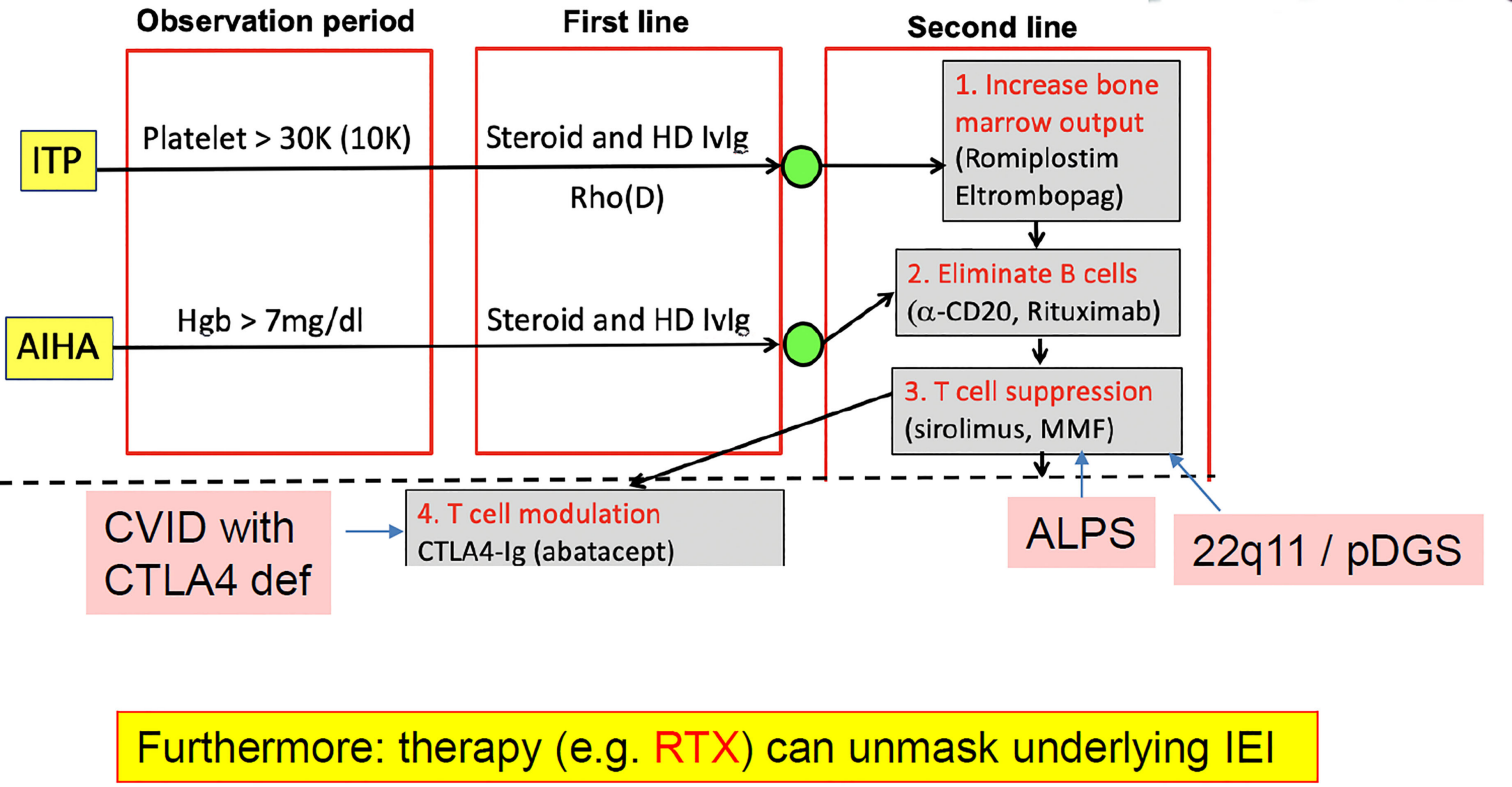
Underlying primary immunodeficiency (PID) in autoimmune cytopenia: early diagnosis can alter the treatment pathway. AIHA, autoimmune hemolytic anemia; ALPS, autoimmune lymphoproliferative syndrome; CVID, common variable immune deficiency; HD, high dose; Hgb, hemoglobin; IEI, inborn error of metabolism (=primary immunodeficiency); ITP, immune thrombocytopenia; IVIg, intravenous immunoglobulin; RTX, rituximab.

As mentioned, it is important to know about underlying PID in patients with autoimmune cytopenia because it can determine the most appropriate treatment. The process for managing patients with ITP or autoimmune hemolytic anemia includes first-line therapy with high-dose steroids and/or immunoglobulin for acute disease, followed by close monitoring for relapse. If the patient is asymptomatic and the platelet, red blood cell or neutrophil count is not critical, the patient is generally followed for a period of observation. If counts are low, second-line therapy is initiated. The second-line phase involves three possible steps: (1) starting measures to increase bone marrow output, (2) elimination of B cells, and (3) suppression of T-cell activation (**Figure 3**). The selection of second-line therapy is often empirical; however, in the case of PIRD and PID, a more targeted therapy can be achieved. For example, CTL4-Ig in CTLA4 deficiency, m-TOR blockade in ALPS, or PI3K inhibition in APDS (45).

**Figure 4:**
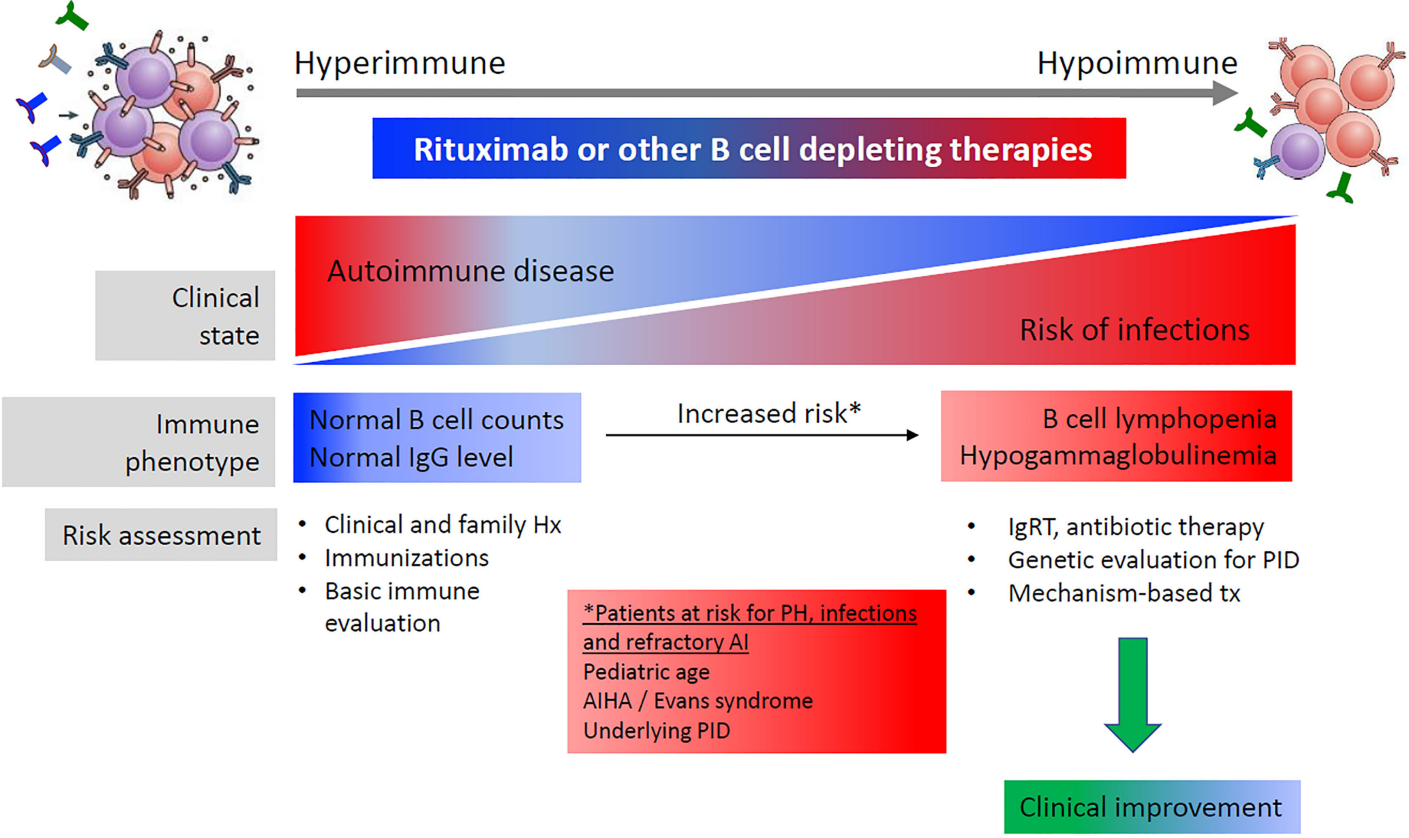
B cell-related antibody responses to B cell-depleting therapy. Reproduced with permission from. (10)

Furthermore, prolonged hypogammaglobulinemia after therapy with rituximab can itself be a pointer towards an underlying PID (55). A review of publications where hypogammaglobulinemia, infections and PID were diagnosed after rituximab therapy in patients with non-hematological malignant disease found that the prevalence of hypogammaglobulinemia varied depending on the underlying disorder, and that a subset of cases had prolonged hypogammaglobulinemia and were diagnosed with CVID/PID later in life (10). A recent study of pediatric patients treated with rituximab identified several potential risk factors for the subsequent development of prolonged hypogammaglobulinemia with an associated risk of infection, including low IgG or IgA, which could alert physicians to the possibility of immunodeficiency (9). It was noted that many PID patients had normal IgG before rituximab, and the underlying disease was unmasked by rituximab therapy (**Figure 4**).

In summary, the following factors may help clinicians to differentiate PID from SID in patients with autoimmune disorders. PID patients tend to have: (1) a clinical history of multiple autoimmune manifestations, progression with age, and a treatment-refractory course (rituximab therapy); (2) both infectious and non-infectious complications; (3) a family history of members with variable levels of immune dysregulation, with this being especially telling in cases of AD-inherited gain-of-function variants. As for the diagnostic approach: (1) basic immune phenotyping of immunoglobulins should be performed, while bearing in mind that a normal IgG can be falsely reassuring because low IgG is common in CVID/CID whereas normal IgG may be seen in ALPS and IPEX-like PIRDs; (2) genetic screening is of high importance; (3) investigation of biomarkers for diagnosis and treatment response may suggest an underlying PIRD. Targeted therapy to control immune dysregulation is preferred. This may serve as a bridge to hematopoietic stem-cell transplantation unavoidable in selected cases.

To ensure favorable clinical outcomes, a multidisciplinary, potentially multicenter, approach is needed for both pediatric and adult patients. This may involve hematology, bone marrow transplant, pulmonology, rheumatology, and gastrointestinal departments, with representation from pediatric and adult departments.

### Treatment Approach for Antibody Deficiency in SID vs PID Patients

The European expert consensus group has issued recommendations for starting IgRT in patients with SID and hematological malignancy, based on several parameters including hypogammaglobulinemia, infections and vaccine responses (56). The criteria and associated algorithm are similar to those used in patients with PID for the initiation of IgRT. It would be helpful to be able to predict which patients with lymphoproliferative diseases are likely to develop SID and infection and need IgRT. Recently, practical guidance for the diagnosis and management of secondary hypogammaglobulinemia has been produced by the AAAAI Primary Immunodeficiency and Altered Immune Response Committees (55). This in-depth report discusses the fact that there are no definitive guidelines on when to start IgRT, and highlights the importance of age-appropriate reference ranges for pediatric patients, and stratification of low IgG levels (<700 mg/dL, 400–699 mg/dL, 200–399 mg/dL, and 0–199 mg/dL) and length of hypogammaglobulinemia to determine timing for IgRT (55).

An example scenario is CLL, hypogammaglobulinemia and risk of infections. A group in Denmark has looked at the factors that predict SID in CLL, and whether there are biomarkers that can help determine when to initiate IgRT (57). They developed a CLL Treatment-Infection Model (CLL-TIM) that predicted which patients were at high risk of treatment and/or infection within the 2 years after diagnosis. Machine learning algorithms were developed based on data from 4149 patients, and incorporating 13 baseline tests, 216 diagnoses, 153 pathology codes, 46 microbiologic findings, and 183 routine laboratory tests. Patients were classified as high confidence/high risk, low confidence, and high confidence/low risk for infection. The model did not provide information regarding immunoglobulins, which is in contrast to other studies which have found that significant hypogammaglobulinemia (IgG <4g/L) or low IgG and IgA are associated with the risk of serious infection (58, 59). Using the CLL-TIM algorithms, the occurrence of infections several years prior to the diagnosis of CLL was highly specific for the risk of infection after diagnosis but prior to CLL treatment. The authors concluded that personalized risk factors can aid efforts aimed at personalized treatment for patients with CLL, such as the use of immunoglobulin (57).

## Discussion

SID and PID crossovers present a challenge for accurate diagnosis, and therefore appropriate treatment. It is important to consider the possibility of immune deficiency in patients with hematological malignancy or autoimmune disorders. Early recognition of PID will enable patients to receive the most appropriate therapy at a stage when end-organ damage can be avoided. Clinicians therefore need to adopt a broad perspective: in addition to a history of recurrent infection, evidence of immune dysregulation, syndromic features, malignancy, and family history, can point towards PID.

Patients with hematological cancer and immune deficiency are at high risk of a worse prognosis due to complications such as infection, and should be screened for PID as well as SID. A combined approach for early PID screening at the time of diagnosis of a lymphoproliferative syndrome has prognostic implications and can direct counselling for the patient and their family. In suspected cases, upfront genetic studies at the time of lymphoproliferative syndrome diagnosis are clinically relevant. There are currently no consensus guidelines for cancer screening and follow-up in patients with PID, a group known to be at increased risk of cancer. There is a need for international guidelines for malignancy screening and management in patients with PID.

Underlying PID is common in the pool of patients who have autoimmune cytopenia. Therapy differs depending on the cause of the cytopenia. Moreover, treatment with immune-modulating drugs such as rituximab or checkpoint inhibitors can reveal the presence of PID. Therefore, it is important that physicians managing patients with autoimmune cytopenia consider the possibility of PID.

A detailed immune work-up is important for the diagnosis of PIDs, as well as monitoring biomarkers that may reflect response to immune modulation (CD19^hi^21^low^ B cells [age-associated B cells] and Tfh cells (60) (**Table 3**). It is also relevant in patients without a genetic diagnosis in order to identify any secondary immune dysfunction. Other authors have suggested immune profile-based scoring systems could be helpful (60).

Healthcare professionals treating patients with immunodeficiencies, and those working in departments such as hematology or oncology where some of the manifestations may first be diagnosed, need to be aware of the possibility of PID as well as SID. An interdisciplinary approach is needed to provide early diagnosis and appropriate management of PID and SID in patients with hematological malignancy or autoimmune disorders.

In summary, the highly variable presentation of PIDs mean that antibody deficiencies initially attributed to SID due to underlying disease and/or therapeutic agents used to treat hematological malignancies and autoimmune disorders, may actually be due to an underlying PID. Physicians need to be aware of the possibility of SID and PID crossovers in patients with hematological malignancies or autoimmune disorders.

## Author Contributions

All authors contributed extensively to the work presented in this paper. All authors have contributed significantly to the conception, design, or acquisition of data, or analysis and interpretation of data. All authors have participated in in drafting, reviewing, and/or revising the manuscript and have approved its submission.

## Funding

This project was funded by Grifols, during the Meeting of The European Society for Immunodeficiencies (ESID 2021).

## Conflict of Interest

MB has served on the advisory boards of Grifols, CSL Behring and Takeda. He is on the speaker program for Grifols and CSL Behring. He is a consultant for UpToDate and the Immune Deficiency foundation. He serves on the data safety monitoring boards for Green Cross, Alladept and GSK.

SS-R has served on advisory boards for Grifols, CSL Behring, Takeda, Octapharma, Biotest and Biogen.

JEW has received grant/research/clinical trial support from Takeda, Janssen, Chiesi, MustangBio, ADMA Biologicals, Octapharma; has been consultant/ participate in Advisory Boards for Takeda, X4- Pharmaceuticals, CSL-Behring, Grifols, ADMA Biologicals, Enzyvant, Regeneron; has participated in Speaker's Bureau for Takeda.

## Publisher’s Note

All claims expressed in this article are solely those of the authors and do not necessarily represent those of their affiliated organizations, or those of the publisher, the editors and the reviewers. Any product that may be evaluated in this article, or claim that may be made by its manufacturer, is not guaranteed or endorsed by the publisher.
